# 5-Diethyl­amino-2-{[2-(2,4-dinitro­phen­yl)hydrazin-1-yl­idene]meth­yl}phenol

**DOI:** 10.1107/S1600536810044983

**Published:** 2010-11-10

**Authors:** Lin-xiu Zhao, Gang-shen Li

**Affiliations:** a College of Chemical Engineering and Environment, North University of China, Taiyuan 030051, People’s Republic of China; b Key Laboratory of Surface and Interface Science of Henan, School of Material & Chemical Engineering, Zhengzhou University of Light Industry, Zhengzhou 450002, People’s Republic of China

## Abstract

In the title compound, C_17_H_19_N_5_O_5_, obtained from the condensation reaction of 4-diethyl­amino-2-hy­droxy­benzalde­hyde and 2,4-dinitro­phenyl­hydrazine, the two benzene rings are twisted by a dihedral angle of 1.75 (12)°. The nitro groups are slightly twisted with the respect to the benzene ring to which they are attached, making dihedral angles of 8.20 (15) and 5.78 (15)°. An intra­molecular O—H⋯N hydrogen bond occurs. In the crystal, mol­ecules are linked by pairs of inter­molecular N—H⋯O hydrogen bonds, forming dimers through *R*_2_^2^(12) rings. These dimers are further linked by C—H⋯O and C—H⋯π and weak slipped π–π inter­actions [centroid–centroid distance = 3.743 (2)Å]. One of the ethyl groups is disordered over two positions, with occupancy factors in the ratio 0.72:0.28.

## Related literature

For related structures, see: Baughman *et al.* (2004 ▶); Kuleshova *et al.* (2003 ▶); Ohba (1996 ▶); Okabe *et al.* (1993 ▶); Szczesna & Urbanczyk-Lipkowska (2002 ▶); Zhen & Han (2005 ▶). For discussion of hydrogen-bonding patterns, see: Etter *et al.* (1990 ▶); Bernstein *et al.* (1995 ▶). 
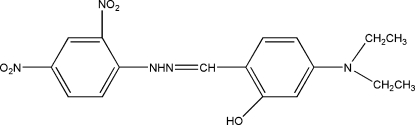


## Experimental

### 

#### Crystal data

C_17_H_19_N_5_O_5_
*M* *_r_* = 373.37
Triclinic, 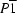

*a* = 8.5300 (7) Å
*b* = 8.5410 (4) Å
*c* = 12.4910 (11) Å
α = 84.554 (7)°
β = 89.733 (6)°
γ = 75.109 (7)°
*V* = 875.31 (11) Å^3^
*Z* = 2
Mo *K*α radiation
μ = 0.11 mm^−1^
*T* = 293 K
0.22 × 0.19 × 0.17 mm

#### Data collection

Bruker SMART CCD area-detector diffractometer
Absorption correction: multi-scan (*SADABS*; Bruker, 1998 ▶) *T*_min_ = 0.975, *T*_max_ = 0.980
5395 measured reflections
3069 independent reflections
1727 reflections with *I* > 2σ(*I*)
*R*_int_ = 0.033

#### Refinement

*R*[*F* ^2^ > 2σ(*F* ^2^)] = 0.055
*wR*(*F* ^2^) = 0.125
*S* = 0.95
3069 reflections
257 parameters
1 restraint
H-atom parameters constrained
Δρ_max_ = 0.18 e Å^−3^
Δρ_min_ = −0.19 e Å^−3^

### 

Data collection: *SMART* (Bruker, 1998 ▶); cell refinement: *SAINT* (Bruker, 1998 ▶); data reduction: *SAINT*; program(s) used to solve structure: *SHELXTL* (Sheldrick, 2008 ▶); program(s) used to refine structure: *SHELXTL*; molecular graphics: *ORTEPIII* (Burnett & Johnson, 1996 ▶), *ORTEP-3 for Windows* (Farrugia, 1997 ▶) and *PLATON* (Spek, 2009 ▶); software used to prepare material for publication: *SHELXTL*.

## Supplementary Material

Crystal structure: contains datablocks global, I. DOI: 10.1107/S1600536810044983/dn2616sup1.cif

Structure factors: contains datablocks I. DOI: 10.1107/S1600536810044983/dn2616Isup2.hkl

Additional supplementary materials:  crystallographic information; 3D view; checkCIF report

## Figures and Tables

**Table 1:** Hydrogen-bond geometry (Å, °) *Cg*2 is the centroid of the C8–C13 ring.

*D*—H⋯*A*	*D*—H	H⋯*A*	*D*⋯*A*	*D*—H⋯*A*
O1—H1⋯N1	0.82	1.95	2.672 (3)	146
N2—H2⋯O3^i^	0.86	2.51	3.344 (3)	162
C15—H15*B*⋯O4^ii^	0.96	2.43	3.359 (6)	164
C14—H14*C*⋯*Cg*2^iii^	0.96	2.71	3.620 (4)	157

Symmetry codes: (i) 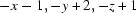; (ii) 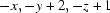; (iii) 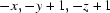.

**Fig. 1.**
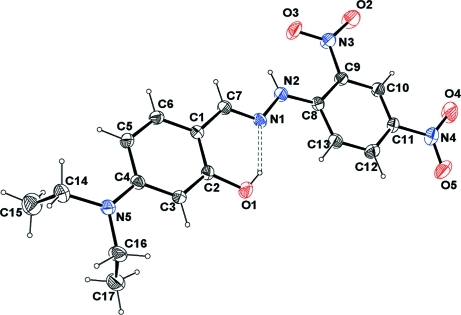
Molecular view of I with the atom labeling scheme. Displacement ellipsoids are drawn at the 30% probability level. Only the major component of the disordered ethyl group is shown. H atoms are represented as small spheres of arbitrary radii. Intramolecular H bond is shown as dashed line.

**Fig. 2.**
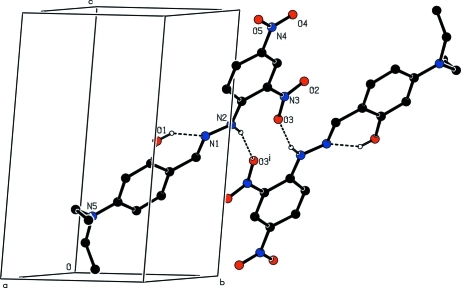
Partial packing view showing the formation of the R22(12) ring. H atoms not involved in the hydrogen bondings have been omitted for clarity. [Symmetry codes: (i) -x-1, -y+2, -z+1]

## supplementary crystallographic information

### Comment

2,4-Dinitrophenylhydrazine is a reagent which is widely used for condensation with aldehydes and ketones. Several phenylhydrazone derivatives have been shown to be potentially DNA-damaging and are mutagenic agents(Okabe *et al.* 1993). Structural information for phenylhydrazone derivatives is useful in studying their coordination properties. As part of our work, we have synthesized the title compound and report the crystal structure.

The molecule is coplanar, the two phenyl rings are only twisted by a dihedral angle of 1.75 (12)°. Bond lengths and bond angles agree with those of other dinitrophenylhydrazone derivatives(Ohba,1996; Baughman *et al.*, 2004; Kuleshova *et al.*, 2003; Szczesna & Urbanczyk-Lipkowska, 2002; Zhen & Han, 2005)

There are intramolecular N—H···O hydrogen bond within the hydrazone molecules. Molecules are linked two by two by intermolecular N—H···O hydrogen bonds (Table 1, Fig. 1) which form a R_2_^2^(12) ring (Etter *et al.*, 1990; Bernstein *et al.*, 1995). These dimer are further linked by C-H···O, C-H···π (Table 1) and by weak slippest π-π interactions [centroid to centroid = 3.743 (2)Å, interplanar distance = 3.42Å and offset angle= 24°]

### Experimental

2,4-dinitrophenylhydrazine (1 mmol, 0.198 g) was dissolved in anhydrous ethanol (10 ml), H_2_SO_4_(98%, 0.5 ml) was then added and The mixture was stirred for several minitutes at 351k, 4-(diethylamino)-2-hydroxybenzaldehyde (1 mmol, 0.193 g) in ethanol (10 mm l) was added dropwise and the mixture was stirred at refluxing temperature for 2 h. The product was isolated and recrystallized from DMF, red single crystals of (I) was obtained after one month.

### Refinement

All H atoms were positioned geometrically and refined as riding with C—H=0.93 (aromatic), 0.97(methylene), 0.96 Å(methyl) and N—H=0.86 Å, with *U*_iso_(H)=1.2*U*_eq_(CH, CH_2_ or NH) and *U*_iso_(H)=1.5*U*_eq_(CH_3_).

The C15 atom is distributed over two positions C15 and C15B. The occupancy factor with the sum of the occupancy factor constraints to be 1.0, was first refined using a overall isotropic thermal parameter for the two carbon atoms. Once the occupancy factor has been determined, it was fixed and the isotropic thermal parameters were freely refined. The geometry of the ethyl has been kept chemically reasonable using restraints (SAME, Sheldrick, 2008). Using such disoredered model improved greatly the refinement.

### Crystal data

**Table:**

C_17_H_19_N_5_O_5_	*Z* = 2
*M**_r_* = 373.37	*F*(000) = 392
Triclinic, *P*1	*D*_x_ = 1.417 Mg m^−^^3^
Hall symbol: -P 1	Mo *K*α radiation, λ = 0.71073 Å
*a* = 8.5300 (7) Å	Cell parameters from 2780 reflections
*b* = 8.5410 (4) Å	θ = 3.0–25.0°
*c* = 12.4910 (11) Å	µ = 0.11 mm^−^^1^
α = 84.554 (7)°	*T* = 293 K
β = 89.733 (6)°	Block, red
γ = 75.109 (7)°	0.22 × 0.19 × 0.17 mm
*V* = 875.31 (11) Å^3^	

### Data collection

**Table:**

Bruker SMART CCD area-detector diffractometer	3069 independent reflections
Radiation source: fine-focus sealed tube	1727 reflections with *I* > 2σ(*I*)
graphite	*R*_int_ = 0.033
ω scans	θ_max_ = 25.0°, θ_min_ = 3.0°
Absorption correction: multi-scan (*SADABS*; Bruker, 1998)	*h* = −10→10
*T*_min_ = 0.975, *T*_max_ = 0.980	*k* = −10→9
5395 measured reflections	*l* = −14→14

### Refinement

**Table:**

Refinement on *F*^2^	Primary atom site location: structure-invariant direct methods
Least-squares matrix: full	Secondary atom site location: difference Fourier map
*R*[*F*^2^ > 2σ(*F*^2^)] = 0.055	Hydrogen site location: inferred from neighbouring sites
*wR*(*F*^2^) = 0.125	H-atom parameters constrained
*S* = 0.95	*w* = 1/[σ^2^(*F*_o_^2^) + (0.0449*P*)^2^] where *P* = (*F*_o_^2^ + 2*F*_c_^2^)/3
3069 reflections	(Δ/σ)_max_ = 0.001
257 parameters	Δρ_max_ = 0.18 e Å^−^^3^
1 restraint	Δρ_min_ = −0.19 e Å^−^^3^

### Special details

**Table:**

Geometry. All esds (except the esd in the dihedral angle between two l.s. planes) are estimated using the full covariance matrix. The cell esds are taken into account individually in the estimation of esds in distances, angles and torsion angles; correlations between esds in cell parameters are only used when they are defined by crystal symmetry. An approximate (isotropic) treatment of cell esds is used for estimating esds involving l.s. planes.
Refinement. Refinement of *F*^2^ against ALL reflections. The weighted *R*-factor *wR* and goodness of fit *S* are based on *F*^2^, conventional *R*-factors *R* are based on *F*, with *F* set to zero for negative *F*^2^. The threshold expression of *F*^2^ > σ(*F*^2^) is used only for calculating *R*-factors(gt) *etc*. and is not relevant to the choice of reflections for refinement. *R*-factors based on *F*^2^ are statistically about twice as large as those based on *F*, and *R*- factors based on ALL data will be even larger.

### Fractional atomic coordinates and isotropic or equivalent isotropic displacement parameters (Å^2^)

**Table:**

	*x*	*y*	*z*	*U*_iso_*/*U*_eq_	Occ. (<1)
O1	0.25187 (19)	0.6271 (3)	0.50881 (14)	0.0542 (6)	
H1	0.1746	0.6862	0.5369	0.081*	
O2	−0.5619 (2)	1.2021 (3)	0.72130 (18)	0.0859 (9)	
O3	−0.4850 (2)	1.0820 (3)	0.57933 (16)	0.0674 (7)	
O4	−0.1960 (3)	1.2721 (3)	0.98125 (17)	0.0822 (8)	
O5	0.0601 (3)	1.1684 (3)	0.96747 (16)	0.0757 (7)	
N1	−0.0540 (2)	0.8040 (3)	0.52221 (16)	0.0361 (5)	
N2	−0.1829 (2)	0.9124 (3)	0.56372 (15)	0.0380 (6)	
H2	−0.2768	0.9352	0.5326	0.046*	
N3	−0.4544 (3)	1.1282 (3)	0.6656 (2)	0.0510 (7)	
N4	−0.0803 (3)	1.1929 (3)	0.93582 (19)	0.0538 (7)	
N5	0.3830 (2)	0.2866 (3)	0.22459 (17)	0.0418 (6)	
C14	0.3429 (3)	0.2320 (4)	0.1233 (2)	0.0612 (9)	0.72
H14A	0.4100	0.1223	0.1194	0.073*	0.72
H14B	0.2312	0.2253	0.1268	0.073*	0.72
C15	0.3598 (5)	0.3248 (6)	0.0249 (3)	0.0738 (14)	0.72
H15A	0.4711	0.3271	0.0167	0.111*	0.72
H15B	0.2932	0.4340	0.0258	0.111*	0.72
H15C	0.3263	0.2760	−0.0342	0.111*	0.72
C14B	0.3429 (3)	0.2320 (4)	0.1233 (2)	0.0612 (9)	0.28
H14C	0.2653	0.1684	0.1407	0.073*	0.28
H14D	0.2842	0.3292	0.0796	0.073*	0.28
C15B	0.4529 (12)	0.1441 (16)	0.0563 (9)	0.067 (3)	0.28
H15D	0.4003	0.1415	−0.0110	0.101*	0.28
H15E	0.4944	0.0350	0.0892	0.101*	0.28
H15F	0.5407	0.1944	0.0441	0.101*	0.28
C1	0.0349 (3)	0.6209 (3)	0.38800 (18)	0.0316 (6)	
C2	0.1988 (3)	0.5694 (3)	0.42192 (19)	0.0347 (7)	
C3	0.3117 (3)	0.4608 (3)	0.36813 (19)	0.0372 (7)	
H3	0.4188	0.4291	0.3931	0.045*	
C4	0.2693 (3)	0.3972 (3)	0.27695 (19)	0.0340 (6)	
C5	0.1057 (3)	0.4485 (3)	0.24127 (19)	0.0378 (7)	
H5	0.0733	0.4094	0.1803	0.045*	
C6	−0.0054 (3)	0.5558 (3)	0.29623 (19)	0.0378 (7)	
H6	−0.1127	0.5868	0.2715	0.045*	
C7	−0.0867 (3)	0.7351 (3)	0.4408 (2)	0.0353 (7)	
H7	−0.1934	0.7603	0.4152	0.042*	
C8	−0.1625 (3)	0.9827 (3)	0.65262 (19)	0.0325 (6)	
C9	−0.2895 (3)	1.0890 (3)	0.7042 (2)	0.0349 (6)	
C10	−0.2620 (3)	1.1567 (3)	0.79614 (19)	0.0388 (7)	
H10	−0.3475	1.2254	0.8288	0.047*	
C11	−0.1096 (3)	1.1223 (3)	0.83856 (19)	0.0379 (7)	
C12	0.0190 (3)	1.0181 (4)	0.7912 (2)	0.0476 (8)	
H12	0.1229	0.9949	0.8213	0.057*	
C13	−0.0069 (3)	0.9507 (3)	0.7016 (2)	0.0420 (7)	
H13	0.0803	0.8811	0.6711	0.050*	
C16	0.5551 (3)	0.2546 (4)	0.2534 (2)	0.0504 (8)	
H16A	0.6138	0.1567	0.2226	0.060*	
H16B	0.5675	0.2336	0.3310	0.060*	
C17	0.6308 (3)	0.3901 (4)	0.2167 (2)	0.0611 (9)	
H17A	0.6177	0.4134	0.1401	0.092*	
H17B	0.7443	0.3582	0.2356	0.092*	
H17C	0.5790	0.4856	0.2508	0.092*	

### Atomic displacement parameters (Å^2^)

**Table:**

	*U*^11^	*U*^22^	*U*^33^	*U*^12^	*U*^13^	*U*^23^
O1	0.0419 (11)	0.0745 (17)	0.0458 (12)	−0.0051 (11)	−0.0017 (9)	−0.0321 (11)
O2	0.0393 (11)	0.118 (2)	0.0921 (17)	0.0162 (12)	−0.0029 (11)	−0.0701 (16)
O3	0.0473 (11)	0.0875 (18)	0.0636 (14)	0.0035 (11)	−0.0156 (10)	−0.0452 (13)
O4	0.0799 (16)	0.102 (2)	0.0634 (15)	−0.0050 (14)	0.0036 (13)	−0.0537 (14)
O5	0.0664 (14)	0.102 (2)	0.0637 (14)	−0.0249 (13)	−0.0202 (12)	−0.0256 (14)
N1	0.0338 (12)	0.0365 (14)	0.0357 (12)	−0.0033 (10)	0.0072 (10)	−0.0090 (11)
N2	0.0330 (12)	0.0389 (15)	0.0385 (13)	0.0000 (10)	0.0015 (10)	−0.0117 (11)
N3	0.0367 (13)	0.0542 (18)	0.0583 (16)	0.0033 (12)	−0.0024 (12)	−0.0271 (14)
N4	0.0623 (17)	0.0568 (19)	0.0433 (15)	−0.0149 (14)	−0.0056 (14)	−0.0109 (13)
N5	0.0365 (12)	0.0470 (16)	0.0454 (14)	−0.0113 (11)	0.0070 (11)	−0.0209 (12)
C14	0.0525 (18)	0.078 (3)	0.056 (2)	−0.0138 (17)	0.0129 (16)	−0.0328 (19)
C15	0.084 (3)	0.083 (4)	0.058 (3)	−0.031 (3)	0.006 (2)	0.000 (3)
C14B	0.0525 (18)	0.078 (3)	0.056 (2)	−0.0138 (17)	0.0129 (16)	−0.0328 (19)
C15B	0.057 (7)	0.083 (10)	0.074 (8)	−0.021 (6)	0.036 (6)	−0.061 (7)
C1	0.0313 (14)	0.0314 (16)	0.0318 (14)	−0.0073 (12)	0.0025 (11)	−0.0034 (12)
C2	0.0376 (15)	0.0386 (18)	0.0307 (15)	−0.0125 (13)	0.0020 (12)	−0.0089 (13)
C3	0.0272 (13)	0.0469 (18)	0.0362 (15)	−0.0047 (12)	0.0013 (12)	−0.0107 (13)
C4	0.0352 (14)	0.0326 (17)	0.0364 (15)	−0.0114 (12)	0.0064 (12)	−0.0066 (13)
C5	0.0373 (15)	0.0395 (17)	0.0386 (15)	−0.0096 (13)	0.0012 (12)	−0.0143 (13)
C6	0.0300 (14)	0.0413 (18)	0.0415 (16)	−0.0068 (12)	−0.0020 (12)	−0.0080 (14)
C7	0.0327 (14)	0.0348 (17)	0.0368 (15)	−0.0048 (12)	0.0016 (12)	−0.0068 (13)
C8	0.0365 (15)	0.0282 (16)	0.0312 (14)	−0.0054 (12)	0.0028 (12)	−0.0036 (12)
C9	0.0299 (14)	0.0336 (17)	0.0388 (15)	−0.0027 (12)	−0.0002 (12)	−0.0078 (13)
C10	0.0366 (15)	0.0381 (18)	0.0385 (16)	−0.0017 (13)	0.0024 (13)	−0.0094 (13)
C11	0.0436 (16)	0.0413 (18)	0.0295 (15)	−0.0109 (13)	−0.0022 (13)	−0.0072 (13)
C12	0.0344 (15)	0.058 (2)	0.0458 (17)	−0.0043 (14)	−0.0064 (13)	−0.0065 (16)
C13	0.0356 (15)	0.0456 (19)	0.0414 (16)	−0.0018 (13)	0.0035 (13)	−0.0110 (14)
C16	0.0351 (15)	0.049 (2)	0.066 (2)	−0.0040 (14)	0.0092 (14)	−0.0243 (16)
C17	0.0512 (18)	0.070 (2)	0.068 (2)	−0.0201 (17)	0.0168 (16)	−0.0263 (18)

### Geometric parameters (Å, °)

**Table:**

O1—C2	1.359 (3)	C1—C2	1.408 (3)
O1—H1	0.8200	C1—C7	1.434 (3)
O2—N3	1.226 (3)	C2—C3	1.376 (3)
O3—N3	1.234 (3)	C3—C4	1.395 (3)
O4—N4	1.216 (3)	C3—H3	0.9300
O5—N4	1.223 (3)	C4—C5	1.412 (3)
N1—C7	1.288 (3)	C5—C6	1.369 (3)
N1—N2	1.377 (3)	C5—H5	0.9300
N2—C8	1.344 (3)	C6—H6	0.9300
N2—H2	0.8600	C7—H7	0.9300
N3—C9	1.434 (3)	C8—C13	1.414 (3)
N4—C11	1.457 (3)	C8—C9	1.419 (3)
N5—C4	1.379 (3)	C9—C10	1.381 (3)
N5—C14	1.460 (3)	C10—C11	1.356 (3)
N5—C16	1.462 (3)	C10—H10	0.9300
C14—C15	1.426 (5)	C11—C12	1.391 (4)
C14—H14A	0.9700	C12—C13	1.351 (3)
C14—H14B	0.9700	C12—H12	0.9300
C15—H15A	0.9600	C13—H13	0.9300
C15—H15B	0.9600	C16—C17	1.500 (4)
C15—H15C	0.9600	C16—H16A	0.9700
C15B—H15D	0.9600	C16—H16B	0.9700
C15B—H15E	0.9600	C17—H17A	0.9600
C15B—H15F	0.9600	C17—H17B	0.9600
C1—C6	1.402 (3)	C17—H17C	0.9600
			
C2—O1—H1	109.5	C4—C5—H5	119.9
C7—N1—N2	116.1 (2)	C5—C6—C1	123.1 (2)
C8—N2—N1	120.3 (2)	C5—C6—H6	118.5
C8—N2—H2	119.8	C1—C6—H6	118.5
N1—N2—H2	119.8	N1—C7—C1	122.6 (2)
O2—N3—O3	121.8 (2)	N1—C7—H7	118.7
O2—N3—C9	118.9 (2)	C1—C7—H7	118.7
O3—N3—C9	119.3 (2)	N2—C8—C13	120.1 (2)
O4—N4—O5	123.6 (3)	N2—C8—C9	124.3 (2)
O4—N4—C11	118.6 (3)	C13—C8—C9	115.7 (2)
O5—N4—C11	117.8 (3)	C10—C9—C8	121.9 (2)
C4—N5—C14	121.1 (2)	C10—C9—N3	116.3 (2)
C4—N5—C16	119.8 (2)	C8—C9—N3	121.8 (2)
C14—N5—C16	117.2 (2)	C11—C10—C9	119.6 (2)
C15—C14—N5	119.0 (3)	C11—C10—H10	120.2
C15—C14—H14A	107.6	C9—C10—H10	120.2
N5—C14—H14A	107.6	C10—C11—C12	120.8 (3)
C15—C14—H14B	107.6	C10—C11—N4	119.7 (2)
N5—C14—H14B	107.6	C12—C11—N4	119.6 (2)
H14A—C14—H14B	107.0	C13—C12—C11	120.1 (2)
H15D—C15B—H15E	109.5	C13—C12—H12	120.0
H15D—C15B—H15F	109.5	C11—C12—H12	120.0
H15E—C15B—H15F	109.5	C12—C13—C8	122.1 (3)
C6—C1—C2	116.0 (2)	C12—C13—H13	119.0
C6—C1—C7	120.4 (2)	C8—C13—H13	119.0
C2—C1—C7	123.6 (2)	N5—C16—C17	114.4 (2)
O1—C2—C3	117.4 (2)	N5—C16—H16A	108.7
O1—C2—C1	121.0 (2)	C17—C16—H16A	108.7
C3—C2—C1	121.7 (2)	N5—C16—H16B	108.7
C2—C3—C4	121.5 (2)	C17—C16—H16B	108.7
C2—C3—H3	119.3	H16A—C16—H16B	107.6
C4—C3—H3	119.3	C16—C17—H17A	109.5
N5—C4—C3	121.0 (2)	C16—C17—H17B	109.5
N5—C4—C5	121.4 (2)	H17A—C17—H17B	109.5
C3—C4—C5	117.6 (2)	C16—C17—H17C	109.5
C6—C5—C4	120.1 (2)	H17A—C17—H17C	109.5
C6—C5—H5	119.9	H17B—C17—H17C	109.5
			
C7—N1—N2—C8	175.9 (2)	N1—N2—C8—C9	−176.6 (2)
C4—N5—C14—C15	86.9 (4)	N2—C8—C9—C10	179.3 (2)
C16—N5—C14—C15	−77.4 (4)	C13—C8—C9—C10	0.1 (4)
C6—C1—C2—O1	178.8 (2)	N2—C8—C9—N3	1.3 (4)
C7—C1—C2—O1	−0.2 (4)	C13—C8—C9—N3	−177.9 (2)
C6—C1—C2—C3	−0.3 (3)	O2—N3—C9—C10	−8.1 (4)
C7—C1—C2—C3	−179.3 (2)	O3—N3—C9—C10	174.4 (2)
O1—C2—C3—C4	−178.8 (2)	O2—N3—C9—C8	170.0 (3)
C1—C2—C3—C4	0.3 (4)	O3—N3—C9—C8	−7.5 (4)
C14—N5—C4—C3	−173.9 (2)	C8—C9—C10—C11	0.5 (4)
C16—N5—C4—C3	−10.0 (4)	N3—C9—C10—C11	178.6 (2)
C14—N5—C4—C5	7.0 (4)	C9—C10—C11—C12	−0.8 (4)
C16—N5—C4—C5	170.8 (2)	C9—C10—C11—N4	−179.7 (2)
C2—C3—C4—N5	−179.0 (2)	O4—N4—C11—C10	5.3 (4)
C2—C3—C4—C5	0.2 (4)	O5—N4—C11—C10	−175.0 (2)
N5—C4—C5—C6	178.5 (2)	O4—N4—C11—C12	−173.7 (3)
C3—C4—C5—C6	−0.6 (4)	O5—N4—C11—C12	6.0 (4)
C4—C5—C6—C1	0.6 (4)	C10—C11—C12—C13	0.4 (4)
C2—C1—C6—C5	−0.1 (4)	N4—C11—C12—C13	179.4 (2)
C7—C1—C6—C5	178.9 (2)	C11—C12—C13—C8	0.2 (4)
N2—N1—C7—C1	179.2 (2)	N2—C8—C13—C12	−179.7 (2)
C6—C1—C7—N1	−176.6 (2)	C9—C8—C13—C12	−0.4 (4)
C2—C1—C7—N1	2.3 (4)	C4—N5—C16—C17	−72.3 (3)
N1—N2—C8—C13	2.6 (3)	C14—N5—C16—C17	92.2 (3)

### Hydrogen-bond geometry (Å, °)

**Table:**

Cg2 is the centroid of the C8–C13 ring.

**Table:**

*D*—H···*A*	*D*—H	H···*A*	*D*···*A*	*D*—H···*A*
O1—H1···N1	0.82	1.95	2.672 (3)	146
N2—H2···O3^i^	0.86	2.51	3.344 (3)	162
C15—H15B···O4^ii^	0.96	2.43	3.359 (6)	164
C14—H14C···Cg2^iii^	0.96	2.71	3.620 (4)	157

Symmetry codes: (i) −*x*−1, −*y*+2, −*z*+1; (ii) −*x*, −*y*+2, −*z*+1; (iii) −*x*, −*y*+1, −*z*+1.
